# Livestock herding and Fulani ethnicity are a combined risk factor for development of early adverse reactions to antivenom treatment: Findings from a cross-sectional study in Nigeria

**DOI:** 10.1371/journal.pntd.0009518

**Published:** 2021-08-12

**Authors:** Stefanie K. Menzies, Aniekan O. Thomas, Frank-Leonel Tianyi, Saidu B. Abubakar, Abdulsalami Nasidi, Nandul Durfa, Rohit Patel, Anna Trelfa, David G. Lalloo, Abdulrazaq G. Habib, Robert A. Harrison

**Affiliations:** 1 Centre for Snakebite Research and Interventions, Department of Tropical Disease Biology, Liverpool School of Tropical Medicine, Liverpool, United Kingdom; 2 Centre for Drugs and Diagnostics, Liverpool School of Tropical Medicine, Liverpool, United Kingdom; 3 Kaltungo General Hospital, Kaltungo, Gombe State, Nigeria; 4 Special Projects Unit, Federal Ministry of Health, Abuja, Nigeria; 5 Department of Medicine, Bayero University Kano, Kano, Nigeria; Institut de Recherche pour le Développement, BENIN

## Abstract

**Background:**

Adverse reactions to antivenom considerably complicate the clinical management of snakebite envenomed patients because it necessitates a temporary suspension of life-saving antivenom, increases costs and can compromise patient outcomes. This study sought to explore the association between cattle-herding occupation and ethnic group and the occurrence of early adverse reactions to antivenom.

**Methods:**

This cross-sectional study was conducted between the 25th April and 11th July 2011 at the Kaltungo General Hospital in north east Nigeria. The exposure variable of cattle-herding occupation showed a strong correlation with the ethnic group variable, thus these were combined into a new variable with three categories (Fulani and herder, either Fulani or herder, and neither Fulani nor herder). The outcome variable was the occurrence of early adverse reactions, defined as any new symptoms occurring within 6 hours of antivenom administration. Odds Ratios were estimated using multivariable logistic regression models controlling for potential confounders.

**Results:**

Among 231 envenomed snakebite victims, the overall incidence of early adverse reactions was 11.9% (95% confidence intervals: 8.0–16.9%). Patients who were Fulani and herders had a higher incidence of early adverse reactions compared to patients who were neither Fulani nor herders (20% vs 5.7%). After adjusting for age and gender, victims who were Fulani and herders were 5.9 times more likely to have an early adverse reaction, compared to victims who were neither Fulani nor herders (95% CI: 1.88–18.59; p = 0.002).

**Interpretation:**

To the best of our knowledge, this is the first study to provide evidence of higher odds of early adverse reactions among patients from a particular occupation and/or ethnic group. We recommend that snake envenomed patients of Fulani origin be especially closely monitored for adverse reactions, that hospitals receiving these patients be appropriately resourced to manage both envenoming and adverse reactions and that premedication with adrenaline should be considered. Our findings provide an argument for speculation on the influence of immunological or lifestyle-related differences on the occurrence of early adverse reactions to antivenom.

## Author summary

Antivenom is the first-choice treatment of systemic snake envenoming that annually affects between 1.8–2.7 million victims globally. Access to antivenom is especially poor for those in greatest need because they typically reside in impoverished, rural tropical communities dependent upon health facilities with limited resources. In addition, clinical treatment of snakebite victims is further complicated by early adverse reactions (EARs) to antivenom-treatment. The causes of antivenom-associated EARs are poorly understood and under-researched. Despite antivenom producers instituting costly remedial manufacturing steps (removal of pyrogens and other impurities) to make their products safer, EARs still affect a high proportion of antivenom-treated patients. Instigated by anecdotal observations to the corresponding author from clinicians in rural Nigerian hospitals that snakebite victims of cattle-herding occupation, and especially those of Fulani ethnicity, suffer more frequent EARs than other groups, this cross-sectional study identified that risks of developing EARs to antivenom treatment include the ethnicity and pastoral lifestyle of snakebite patients. To our knowledge, this is the first study to identify that EARs to antivenom-treatment include factors associated with the victim, as well as factors related to the antivenom itself. We emphasise the need for more research on the causes of adverse reactions to antivenom so that strategies to reduce incidence can be implemented.

## Introduction

Snakebite envenoming kills between 81,000–138,000 victims annually and between 1.8–2.7 million envenomed victims require treatment [1]. The first-choice treatment for envenoming is antivenom: immunoglobulins purified from the blood of horses or sheep hyper-immunised with venom. Whilst life-saving, antivenom treatment causes adverse reactions in around 20% of patients on average but, depending upon the antivenom brand (total protein content varies by manufacturer), this can be as high as 88% [2]. Whilst common, adverse reactions to antivenom are often undisclosed in publications of clinical studies, as demonstrated by Potet *et al*’s review of clinical evidence for antivenom effectiveness in sub-Saharan Africa [3], which highlighted the value of this information to clinicians treating snakebite patients. Furthermore, a lack of consistency in definitions used to categorise severity of adverse reactions to antivenom impedes comparison between the safety profiles of different antivenom products.

Adverse reactions to antivenom considerably complicate the clinical management of patients because it requires a temporary cessation of antivenom administration, additional medicines, resuscitation equipment and clinical expertise that are often unavailable in rural health facilities closest to most snakebite victims, and increases costs to both patients and health infrastructures. Clinicians apprehensive of adverse reactions have referred patients to other facilities, delayed administering antivenom or given suboptimal doses, which can be detrimental to patient outcomes [4].

Adverse reactions to antivenom are classified into three groups:

1. Early adverse reactions occur within 24 hours of antivenom administration [2], and typically manifest within the first three hours [5]. Most patients experience mild to moderate symptoms such as skin reactions (urticaria and pruritus) and gastrointestinal disturbances (vomiting, nausea, colic, diarrhoea). Severe anaphylactic reactions presenting as bronchospasm, angioedema or hypotension are less frequent but can be fatal. The true incidence of fatal reactions is unknown, under-researched, under-reported and can be mis-attributed to venom pathology [5].
2. Pyrogenic reactions typically occur in the first two hours after antivenom administration and present as fever, chills, headache, myalgia, tachycardia, vasodilatation and hypotension [2]. These are caused by contamination of antivenom during manufacture with pyrogenic substances, such as bacterial endotoxins, protein aggregates and dimers [2,6].
3. Delayed or late adverse reactions (‘serum sickness’) occur between five to fourteen days after antivenom administration and are characterised by symptoms consistent with serum sickness observed with other antisera therapies [6]. These symptoms include gastrointestinal disturbances (nausea, diarrhoea, vomiting), urticaria, myalgia, arthralgia, pruritic rashes and lymphadenopathy [4]. Delayed serum sickness reactions to antivenom are an immunoglobulin-mediated response to exogenous immunoglobulins which activate the complement pathway [2]. Clinical evidence has shown that late adverse reactions are more likely to occur in patients previous administered serotherapy and given higher doses of antivenom [6].

Early adverse reactions are the most common category of adverse reactions and they have the greatest impact on patient care, as treatment is usually stopped following an early adverse reaction. Research evidence on the cause of these reactions is equivocal. Hypotheses include an IgE-mediated Type I hypersensitivity reaction in patients with prior exposure or sensitisation to antivenom components [7] or other animal-derived immunoglobulins (i.e. anti-tetanus and anti-rabies serotherapies), and a non-IgE-mediated activation of the complement system or heterophilic antibodies among patients without prior exposure [2,8].

There is a dearth of evidence on risk factors of early adverse reactions. Anecdotal observations from clinicians in North East Nigeria to the corresponding author suggested that cattle herders, particularly of the Fulani ethnic group, experience frequent early adverse reactions compared to the rest of the population. The Fulani are a major ethnic group in Nigeria, with an estimated population of 15.3 million, and are recognised as the dominant pastoral group in Nigeria [9], whose lifestyle, livelihood and diet are centred on cattle [10]. Fulani pastoralists generate most of their income through the sale of livestock, crops and milk [9]. This study sought to explore the association between cattle herding as an occupation, being a member of the Fulani ethnic group and the occurrence of early adverse reactions to antivenom.

## Methods

### Ethics statement

The study design and ethical considerations were approved by the Research Ethics Committee of the Liverpool School of Tropical Medicine on April 11, 2011 (reference number 11.31LT) and from competent authorities of Kaltungo General Hospital on April 8, 2011 (reference number GHK/ADM/58/VOL.I). Written informed consent was obtained from the participants prior to data collection. Translated versions of the consent forms were used as required, and the information sheet was read out to illiterate patients in their local language, with a thumb print used to signify consent.

### Study population/setting

This cross-sectional prospective study was conducted at the Kaltungo General Hospital (KGH), in the savannah area of Gombe State, Nigeria. Most of the snakebite patients admitted to the hospital are envenomed by the West African saw-scaled viper (*Echis ocellatus*). Many patients bring the dead snake for identification and a 20-minute whole blood clotting test (20WBCT) is performed on all patients to identify patients with incoagulable blood–a distinctive symptom of envenoming by this snake in this region of Nigeria.

Patients were enrolled between 25th April and 11th July 2011. Patients were included if they were aged 18 years or older at the time of admission, they had a positive 20WBCT, they presented within 72 hours of the snakebite and they provided consent to participate in the study. Patients were excluded if they were aged 17 years and under, they had been administered antivenom at another hospital for the current snakebite, they were pregnant, or they had a severe underlying medical condition. All patients were treated with either EchiTAb-G (*E*. *ocellatus* monospecific, intact immunoglobulin antivenom manufactured from immunoglobulins of sheep hyper-immunised with this venom, by MicroPharm Ltd, Wales, UK) or EchiTAb-Plus-ICP (*E*. *ocellatus*, *Bitis arietans*, *Naja nigricollis* tri-valent, intact immunoglobulin antivenom manufactured from immunoglobulins of horses hyper-immunised with these three venoms, by Instituto Clodomiro Picado, Costa Rica) according to local guidelines, irrespective of whether or not they were included in the study.

Following enrolment into the study, patients or their relatives were questioned by the study investigators to collect demographic data (ethnicity, age, occupation, and gender) and self-reported snakebite history (previous snakebite, antivenom received, and adverse reaction). Data was manually recorded prior to transcription onto a secure electronic database. Clinical observations and treatments (antivenom given and dose, observation of adverse reactions and symptoms) were recorded by the investigators and transcribed into the database.

### Exposure variables

The exposure variables were cattle-herding occupation and Fulani or non-Fulani ethnicity. In north-eastern Nigeria, ethnicity and occupation are strongly correlated, especially for people of Fulani ethnicity. Exploratory analysis of these variables showed a strong correlation in our dataset (r = 0.5). Consequently, both variables were collapsed into binary variables, and a new variable with three categories (Fulani and livestock herder, either Fulani or herder, and neither Fulani nor herder) was created to address this collinearity.

### Outcome variable

The outcome variable was the occurrence of early adverse reactions among patients treated with antivenom. Prior evidence from a randomised controlled clinical trial found no significant difference in the risk of adverse reactions after treatment with the monovalent and trivalent EchiTAb antivenoms [11]. Consequently, no distinction between the effects of EchiTAb-G and EchiTAb-Plus-ICP were made in our study. An early adverse reaction was defined as any new non-pyrogenic symptom (pruritus, abdominal pain, nausea, vomiting, bronchospasm, angioedema, or hypotension) occurring within six hours following antivenom administration. The comparison group included patients who had symptoms of later reactions (between 6 hours and three days/discharge) and patients who had no reactions.

### Potential confounding variables

Data was collected on demographic (age and gender) and clinical factors (initial dose, repeated doses, previous snakebite, previous antivenom use) with epidemiological evidence of an effect on the risk of early adverse reactions to antivenom.

### Sample size

For a fixed sample of 231 participants, an early adverse reaction incidence of 22% (the average of previously reported incidence of EAR to EchiTAb-G (18.9%) and EchiTAb-Plus-ICP (25.8%) [11] and type I error rate of 5%, our sample had 90% power to detect an odds ratio of 3.25 between patients who were Fulani and herders and patients who were neither Fulani nor herders.

### Statistical analysis

Descriptive statistics were used to summarise the demographic and clinical characteristics of the study population. Continuous variables were summarised using the median and the interquartile range, while categorical variables were summarised using frequencies and proportions. The incidence of early adverse reactions and the corresponding 95% confidence interval were calculated and presented per group of the derived exposure variable. A univariable analysis explored the association between covariables and the exposure and outcome variables. The Kruskal Wallis test was used to compare continuous and categorical variables, while chi-squared and Fisher’s Exact tests were used for comparing categorical variables. Potential confounders were chosen based on epidemiological and statistical (p < 0.2) bases. Multivariable logistic regression models were built using a stepwise forward regression approach. Age was included a priori into the minimally adjusted model due to strong epidemiological evidence on the association between age and early adverse reactions to antivenom. Crude and adjusted odds ratios were used to describe the association between ethnicity and occupation, and the occurrence of early adverse reactions. A likelihood ratio test was used to assess model fitness. Two-sided p-values < 0.05 were taken to indicate statistical significance. All statistical analyses were carried out using Stata v16 (StataCorp LP, College Station, TX, USA).

## Results

Data from 231 victims of *E*. *ocellatus* (West African saw-scaled viper) envenoming who were treated with EchiTAb-G or EchiTAb-Plus-ICP antivenoms were used in this analysis. The median age was 29.0 (IQR 22.0–40.0), with a majority of victims in the 20–29 year age group. There was a male predominance (78.8%) and farming was the most common occupation (40.7%). Cattle herding was the next most common occupation and 93.6% of herders identified themselves as being of the Fulani ethnic group. Most of the victims were treated with the monovalent antivenom (78.8%) and few patients required more than one dose (5%). Table 1 presents a summary of the demographic and clinical characteristics of the study participants. The proportion of patients who received EchiTAb-G was 78.8%, and 19.9% received EchiTAb-Plus-ICP. The outcome of adverse reactions to antivenom was not significantly different between the two antivenoms (Table 1).

**Table 1:** Sociodemographic and clinical characteristics of patients included in this study.

Patient characteristics	No early adverse reaction	Early adverse reaction	Total (N = 231)	P value
Age, years				0.26
Median (IQR)	30.0 (23.0–40.0)	25.0 (20.0–40.0)	29.0 (22.0–40.0)	
Age in categories				0.28
< 20	16 (8.0%)	2 (7.4%)	18 (7.8%)	
20–29	77 (38.7%)	15 (55.6%)	94 (40.7%)	
30–39	52 (25.1%)	3 (11.1%)	55 (23.8%)	
40–49	24 (12.1%)	5 (18.5%)	29 (12.6%)	
>50	28 (13.6%)	2 (7.4%)	30 (13.0%)	
Gender				0.078
Male	157 (76.4%)	25 (92.6%)	182 (78.8%)	
Female	47 (23.6%)	2 (7.4%)	49 (21.2%)	
Ethnic Group				0.008
Fulani	99 (47.2%)	21 (77.8%)	120 (51.9%)	
Hausa	11 (5.5%)	3 (11.1%)	14 (6.1%)	
Tangale	9 (4.5%)	0 (0.0%)	9 (3.9%)	
Tula	4 (2.0%)	0 (0.0%)	4 (1.7%)	
Other	80 (40.2%)	3 (11.1%)	83 (35.9%)	
Occupation				0.29
Herder	52 (24.1%)	11 (40.7%)	63 (27.3%)	
Farmer	83 (41.7%)	11 (40.7%)	94 (40.7%)	
Self employed	10 (5.0%)	1 (3.7%)	11 (4.8%)	
Student	23 (11.6%)	3 (11.1%)	26 (11.3%)	
Other	32 (15.6%)	1 (3.7%)	33 (14.3%)	
Previous snakebite				0.26
Yes	7 (3.5%)	2 (7.4%)	9 (3.9%)	
No	189 (92.5%)	22 (81.5%)	211(91.3)	
Prior antivenom use				0.14
Yes	4 (2.0%)	2 (7.4%)	6 (2.6%)	
No	191 (93.5%)	22 (81.5%)	213 (92.2%)	
Type of Antivenom				0.99
EchiTAb-G	160 (77.9%)	22 (81.5%)	182 (78.8%)	
EchiTAb-Plus-ICP	41 (20.6%)	5 (18.5%)	46 (19.9%)	
Repeated dose				
Yes	8 (3.5%)	4 (14.8%)	12 (5.2%)	0.027
No	186 (91.5%)	21 (77.8%)	207 (89.6%)	

Data are presented as n(%) unless otherwise specified

Of the 231 patients in this study, 27 had signs of an early adverse reaction following the administration of antivenom, giving an overall incidence of 11.9% (95% confidence intervals [CI]: 8.0–16.9%). The incidence was highest among Fulani herders (20%; [95% CI: 10.4–32.9]) and lowest among victims who were neither Fulani nor herder (5.7%; [95% CI: 2.1–12.1]). Mild early adverse reactions were observed in 63% of these victims, and there was no case of a severe adverse reaction.

From the univariable analysis, there was no significant difference in the median age across categories of the exposure (p = 0.59) and the outcome (p = 0.125). There was a significant, male dominated difference in gender across categories of the exposure (p = 0.007) and the outcome. The remaining variables did not satisfy the statistical criteria for inclusion in the multivariable analysis.

After adjusting for age and gender, antivenom-treated patients who were Fulani and cattle-herders were 5.9 times more likely to have an early adverse reaction to antivenom, compared to patients who were neither Fulani nor herders (95% CI: 1.88–18.59; p = 0.002). Patients who were either Fulani or herders were 3.3 times more likely to have an early adverse reaction compared to patients who were neither (95% CI: 1.07–10.41; p = 0.037). Table 2 shows the multivariable analysis with a sequential adjustment for confounding.

**Table 2:** Multivariable analysis of the association between ethnicity/occupation and the occurrence of early adverse reaction.

Complete Case Analysis^ (N = 214)			Model 1		Model 2		Model 3	
	No EAR 188 (87.85)	EAR 26 (12.15)	OR	95% CI	OR	95% CI	OR	95% CI
Neither Fulani nor herder	95 (95)	5 (5)	Reference		Reference		Reference	
Either Fulani or herder	51 (83.61)	10 (16.39)	3.73*	1.20–11.48	3.56*	1.15–11.02	3.34*	1.07–10.42
Fulani and herder	42(79.25)	11 (20.75)	4.98**	1.63–15.22	4.91**	1.60–15.11	5.91**	1.88–18.59

^: Patients with missing data on ethnicity, occupation, early adverse reactions, age and gender variables were dropped; P < 0.05 *; P < 0.01**; P < 0.001***; Model 1 = Unadjusted model; Model 2 = + age; Model 3 = + gender

## Discussion

This study found that 11.9% of patients of *E*. *ocellatus* envenoming developed an early adverse reaction following antivenom treatment at the Kaltungo General Hospital. This incidence varied from a high of 20% among patients who were Fulani and herders, to a low of 5.7% among patients who were neither Fulani nor herders. Patients who were Fulani and herders were 5.9 times more likely to develop an early adverse reaction to antivenom treatment compared to patients who were neither Fulani nor herders, after accounting for age and gender. This study provides epidemiological evidence of a difference in the occurrence of early adverse reactions among snakebite victims of Fulani ethnicity in Northern Nigeria. Amongst this population, cattle-herders are at a higher risk.

To the best of our knowledge, this is the first study to provide epidemiological evidence of higher odds of early adverse reactions among patients of a particular occupation and/or ethnic background. This confirmed anecdotal reports of a higher incidence of these reactions among Fulani cattle herders. Our findings provide a basis to speculate upon the influence of immunological or lifestyle-related differences on the occurrence of early adverse reactions to antivenom treatment, and builds upon previous observations of significantly different rates of EARs to the same antivenom between urban and rural populations [12].

The Fulani group have been described as having key distinctions in levels of circulating antibodies, antibody receptors and cytokines, which may influence responses to antivenom treatment, perhaps through an atopic causation. Thus, prior studies show that Fulani have higher levels of the inflammatory cytokines IL-6, IL-8 IL-12, IFN-α and IFN-γ from an early age [13]. The Fulani group also have a higher prevalence, when compared to neighbouring ethnic groups, of an IL-4 polymorphism associated with allergy and asthma [14]. The production of IL-4 stimulates a Th2 response and class-switching of IgG1 to IgE [15], consistent with the elevated levels of total IgE observed in the Fulani[16]. Furthermore, a role has been demonstrated for the involvement of IL-4 in acute urticaria[17], whilst IL-6 can be a mediator of pruritis [18]. It seems appropriate to speculate that the elevated levels of total IgE and inflammatory cytokines would place the Fulani at higher risk of adverse reactions to the large amounts of heterologous immunoglobulins administered during antivenom treatment. Indeed, the WHO Guidelines for the Management of Snakebites advises that patients with a history of atopic diseases are at a higher risk of severe adverse reactions to antivenom, and recommends that such patients should be appropriately pre-treated [5]. However, despite several studies reporting immunological differences between the Fulani and neighbouring groups in the context of infectious diseases, there is no data in the literature reporting the prevalence of atopy or other allergic diseases in the Fulani.

Our findings show that being both ethnic Fulani and a cattle herder was associated with higher odds of an early adverse reaction than patients who were neither Fulani nor cattle herder. This suggests that cattle exposure increases the odds of adverse reactions to antivenom within the Fulani ethnic group. The low number of non-Fulani cattle herders precluded the assessment of the cattle herding occupation as a single risk factor in non-Fulani patients. However in Europe, cattle farmers are known to have an increased risk of developing allergic reactions to cattle, with up to 20% of cattle farmers being sensitised to cattle allergens (dander, hair, meat and milk) found throughout cattle stables and farmers’ homes [19]. Fulani cattle herders often keep other livestock in addition to cattle, such as sheep, goats and chickens, which may provide an additional route of sensitisation to animal allergens. To determine the influence of cattle exposure on early adverse reactions to antivenom, a future comparison between Fulani pastoralist cattle herders and other cattle-dependent ethnic groups in Africa, such as the Masai or Turkana in East Africa, would be valuable to determine the relative contribution of cattle exposure, lifestyle and diet.

Another explanation could be sensitisation to the allergenic carbohydrate galactose-alpha-1,3-galactose (αGal) following *Amblyomma spp*. tick bites. This carbohydrate plays a central role in IgE-mediated anaphylaxis upon first exposure to the mouse-human chimeric monoclonal antibody cetuximab in αGal-sensitised patients [20], and in IgE-mediated allergy towards mammalian meat and dairy products [21]. αGal-sensitisation is more prevalent in populations with higher exposure to ticks, such as hunters, forest workers, and those living in rural areas [21]. The Fulani pastoralists commonly remove ticks from their cattle manually, of which *Amblyomma* spp. is one of the three tick genera observed in Fulani cattle [22], thus providing a potential route of αGal-sensitisation in cattle farmers. The allergenic αGal carbohydrate has been detected on numerous antivenoms from multiple animal species in both the whole IgG and Fab formats [23,24], and has been proposed to induce an IgE-mediated adverse reaction to antivenom [23]. The above explanations support the findings in our study and provide justification for further research on potential causes of early adverse reactions to antivenom treatment. Although one small clinical study with low power found no correlation between IgE against αGal with increased risk of EARs to antivenom [25], we believe our findings provide a strong rationale to further investigate the contribution of αGal sensitivity, livestock exposure and tick exposure in the development of EARs to antivenom, in a higher-powered prospective study.

The validity of our findings is supported by the fact that the study was adequately powered to detect a strong association between our exposure and outcome variables. Further, the careful creation of our exposure variable, rigorous ascertainment of the outcomes and the robustness of the statistical analysis in this study reinforces the validity of our findings. The main limitation of this study was the cross-sectional design which limits the possibility of reliably establishing a causal association. There was a possibility of selection bias given that it was a hospital-based study, it excluded patients under the age of 18 and the use of questionnaires may have introduced some recall bias. Finally, our findings are subject to residual confounding because we did not account for all known confounders and other important factors such as time since bite and use of traditional treatment/herbal portions–which could be very high among Fulani.

The outcomes of this study suggest the need for greater clinical research on the triggers and mechanisms of antivenom-induced early adverse reactions. A rigorous clinical immunological study will define the attribution of immunogenetics and lifestyle exposures (including sensitisation to cross-reactive animal allergens and antivenom immunoglobulin allergens) upon adverse reactions to antivenom. The unusually large number of envenomed patients from varied genetic and occupational backgrounds admitted to hospitals in north eastern Nigeria provides an ideal study site for such clinical investigation and the statistical power to calculate odds ratios and detect differences in the incidence of adverse reactions to antivenom. It would be of scientific interest and clinical importance to determine whether other ethnic groups or populations dependent upon livestock agriculture, and other occupations with high levels of animal exposure, experience similarly elevated incidences of adverse reactions to antivenom.

In conclusion, we recommend that health facilities in North Eastern Nigeria take extra precautions when managing snakebite patients who are of the Fulani ethnic group and cattle herders. These patients should be appropriately treated with pre-medication and they should receive early and frequent monitoring for adverse reactions after administration of antivenom. The health facilities should be equipped with trained health care workers, essential medications and medical equipment needed to manage antivenom-induced adverse effects.

## Supporting information

S1 STROBE Checklist STROBE statement checklist for reporting of cross-sectional studies. (DOC)
